# Tubulin and Tubulin Posttranslational Modifications in Alzheimer’s Disease and Vascular Dementia

**DOI:** 10.3389/fnagi.2021.730107

**Published:** 2021-10-29

**Authors:** Estibaliz Santiago-Mujika, Ruth Luthi-Carter, Flaviano Giorgini, Raj N. Kalaria, Elizabeta B. Mukaetova-Ladinska

**Affiliations:** ^1^Department of Neuroscience, Behavior and Psychology, University of Leicester, Leicester, United Kingdom; ^2^Department of Genetics and Genome Biology, University of Leicester, Leicester, United Kingdom; ^3^Translational and Clinical Research Institute, Newcastle University, Newcastle upon Tyne, United Kingdom; ^4^Evington Centre, Leicester General Hospital, Leicester, United Kingdom

**Keywords:** vascular dementia, Alzheimer’s disease, tubulin, posttranslational modification, tubulin code

## Abstract

Alzheimer’s disease (AD) and vascular dementia (VaD) are the two most common forms of dementia in older people. Although these two dementia types differ in their etiology, they share many pathophysiological and morphological features, including neuronal loss, which is associated with the microtubule (MT) destabilization. Stabilization of MTs is achieved in different ways: through interactions with MT binding proteins (MTBP) or by posttranslational modifications (PTMs) of tubulin. Polyglutamylation and tyrosination are two foremost PTMs that regulate the interaction between MTs and MTBPs, and play, therefore, a role in neurodegeneration. In this review, we summarize key information on tubulin PTMs in relation to AD and VaD and address the importance of studying further the tubulin code to reveal sites of potential intervention in development of novel and effective dementia therapy.

## Alzheimer’s Disease and Vascular Dementia

Alzheimer’s disease (AD) and vascular dementia (VaD) are two of the most common types of dementia, representing ∼85% of all dementia cases (Qiu et al., 2009; Iadecola, 2013). In some cases, patients show mixed dementia, where they combine features of AD with ischemic lesions (Iadecola, 2013). Although they have been thoroughly studied, there is no effective treatment to halt or reverse these diseases.

AD is the most common form of dementia and it accounts for more than 70% of all clinically diagnosed dementias (Qiu et al., 2009). Although memory is the main impaired feature, language difficulties, emotional and behavioral changes are also present (Steinberg et al., 2008). The neuropathological hallmarks are amyloid plaques and neurofibrillary tangles (NFTs) (Šimić et al., 2016). Additional neuropathological AD features include dystrophic neurites, astrogliosis, neuronal loss, and cortical atrophy (McKhann et al., 2011; Serrano-Pozo et al., 2011).

Amyloid plaques are extracellular aggregates of insoluble 40 and 42 amyloid-β (Aβ) peptide, that although present in healthy people, are significantly increased in AD patients. On the other hand, NFTs are intraneuronal aggregates of hyperphosphorylated and/or truncated and misfolded tau. Contrary to amyloid plaques, there is a correlation between the burden of NFTs and disease progression, as well as AD clinical symptoms (Giannakopoulos et al., 2009; Suemoto et al., 2017).

VaD is the second most common form of dementia, accounting for ∼15% of all cases. In spite of the number of patients diagnosed and the annual costs for this syndrome being highly significant, VaD has not been as thoroughly studied as AD (Iadecola, 2013).

**FIGURE 1:**
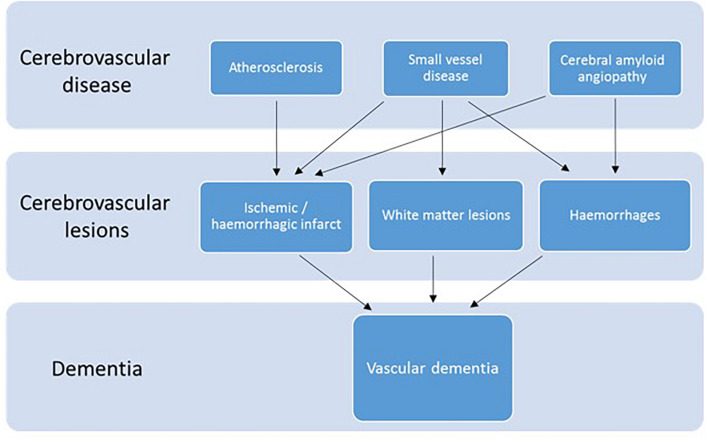
Diagram of the cerebrovascular diseases and lesions that lead to VaD. Modified after (McAleese et al., 2016).

There are four types of VaD: post-stroke dementia, subcortical ischemic vascular dementia, multi-infarct dementia and mixed dementia (Kalaria, 2018; Skrobot et al., 2018). VaD is considered a heterogeneous group of brain disorders since it can be caused by several cerebrovascular pathologies (Iadecola, 2013), such as atherosclerosis, small vessel disease or cerebral amyloid angiopathy (CAA), which is the accumulation of Aβ in vessel walls (Serrano-Pozo et al., 2011). These diseases, in turn, can lead to different cerebrovascular lesions, i.e., ischemic or hemorrhagic infarct, white matter lesions or hemorrhages (McAleese et al., 2016; Smith, 2017; Kalaria, 2018). Although in different ways, these lesions ultimately reduce the blood flow to the brain, and consequently the oxygen supply. The lack of oxygen in the brain is what ultimately leads to vascular dementia (Figure 1).

Although AD and VaD differ in their etiology, they share risk factors, such as age, obesity, the apolipoprotein E4 allele (ApoE ε4 allele) and hypercholesterolemia (Akinyemi et al., 2013). Furthermore, they also share similarities in their pathophysiology (Table 1; Kalaria and Ballard, 1999), i.e., a tendency of Aβ_42_ to be significantly higher in the temporal lobe (Lewis et al., 2006), or a neuronal cell volume loss (Gemmell et al., 2012). In fact, neuronal and synaptic loss are the best predictors of cognitive decline in neurodegenerative diseases (Coleman et al., 2004; Andrade-Moraes et al., 2013; Theofilas et al., 2018). In one study on 14 brains from 80-year-old women, a novel technique called isotropic fractionator was used to determine the absolute cellular composition of brain regions. The study showed there was ∼50% reduction of total neuronal cell numbers in the hippocampus of AD subjects (Andrade-Moraes et al., 2013).

**TABLE 1:** Pathophysiological similarities between AD and VaD.

	Alzheimer’s disease	Vascular dementia
APOE genotype	Higher prevalence of ApoE ε4 allele (Govindpani et al., 2019)	
Altered hemodynamic	Altered vessel hemodynamics, angiogenesis, vascular cell function, vascular coverage, blood-brain barrier permeability. In AD these are attributed to amyloid toxicity (Govindpani et al., 2019)	
Aβ	Significantly higher amounts of Aβ_42_ in the temporal and frontal lobes (Lewis et al., 2006)	Tendency of Aβ_42_ to be higher in the temporal lobe (Lewis et al., 2006)
Tau protein	Significant loss of soluble tau in neocortical areas, hippocampus, and entorhinal cortex (Mukaetova-Ladinska et al., 1993)	Loss of total tau protein in temporal lobe (Mukaetova-Ladinska et al., 2015)
	Widespread significant increase in phosphorylated tau protein (Mukaetova-Ladinska et al., 2015)	No overt change in phosphorylated tau protein (Ser202/Thr205 and Ser262 phosphorylated sites) in temporal and frontal lobes (Mukaetova-Ladinska et al., 2015)
Morphological and cellular changes	Loss of neuronal cell volume (Gemmell et al., 2012)	
	Hippocampal and medial temporal lobe atrophy and CA1 pyramidal neuronal loss (Kril et al., 2002)	
Changes in synaptic proteins	Loss of synaptophysin and SNAP-25 (Mukaetova-Ladinska et al., 2009)	

Not only is neuronal cell death a hallmark of neurodegenerative diseases, but in AD it is also correlated with the severity of the disease (Bussière et al., 2003; Arendt et al., 2015; Martínez-Pinilla et al., 2016; Chi et al., 2018). The same has been found in VaD, where both reduced neuronal volume (Gemmell et al., 2012) and cell counts (Jellinger, 2013) are associated with the severity of cognitive impairment in VaD (Kril et al., 2002; Gemmell et al., 2012).

Although there are several descriptions of mechanisms of cell death (Galluzzi et al., 2018), the major ones linked to neurodegenerative diseases are apoptosis and necrosis (Chi et al., 2018). Apoptosis is a controlled process where there are no spillages of the cell contents to the surrounding, whereas necrosis consists of an uncontrolled cell death, induced by external injury, such as hypoxia or inflammation. Ultimately, the cell membrane breaks and the cellular contents are spilled (D’Arcy, 2019). A new type of controlled necrosis has been recently described, called necroptosis (Degterev et al., 2005). Although similar to necrosis, necroptosis can be activated by death receptors such as TNFR1, which leads to the activation of RIPK1 (Yuan et al., 2019). Furthermore, RIPK1 promotes neuroinflammation, another feature of neurodegenerative diseases (Yuan et al., 2019). Necroptosis has been reported to be activated in AD human brains and positively correlated with Braak stages (Caccamo et al., 2017), with changes in the pathways of apoptosis, autophagy and necrosis depending on the stage of AD (Telegina et al., 2019). Necroptosis has also been reported in the hippocampus of one case of VaD due to ischemic injury. In this case, the necroptosis was related to inflammation and increased cytokines, such as TNF-α and IL-1β (Belkhelfa et al., 2018).

**FIGURE 2:**
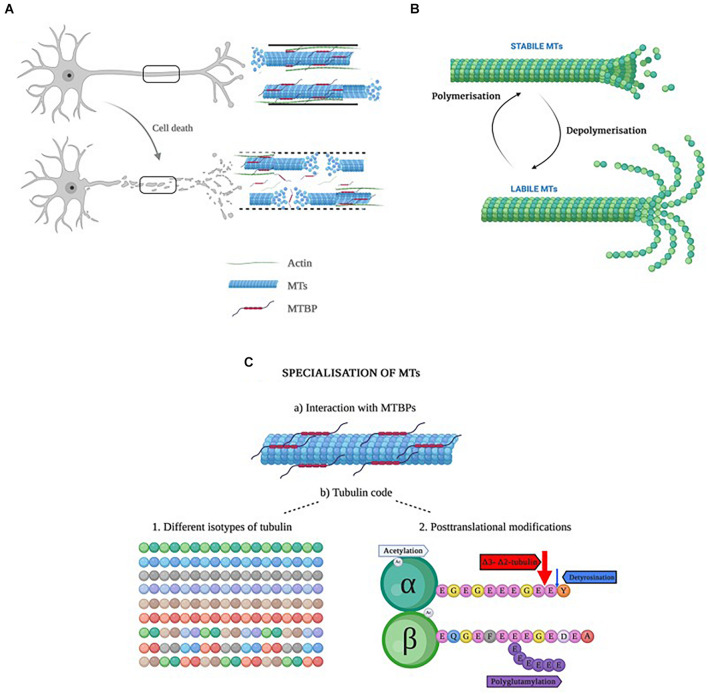
**(A)** Representation of a healthy neuron with a healthy cytoskeleton (top), and a neuron in apoptosis with increased ratio of labile to stabile MTs. **(B)** Representation of the dynamic instability process in microtubules. When MTs are highly dynamic, they are called labile, whereas when the dynamics are slow, they are named stable MTs (Li and Black, 1996). The important aspect of dynamic instability is the presence of stochastic changes between a growth and a shrinkage phase and vice versa. These two phases can occur in parallel in the cells. The dynamic instability is energy-dependent and driven by GTP hydrolysis. **(C)** Schematic representation of the specialization of MTs. Created with BioRender.com.

In apoptosis, the cytoskeleton undergoes significant morphological changes (Bonfoco et al., 1995) due to a disruption of MTs (Liepins and Bustamante, 1994; Figure 2A). Colchicine, an alkaloid plant extract with a therapeutic use in coronary artery diseases, inflammatory and fibrotic conditions, is a drug that disrupts the MTs by fragmenting the tubulin heterodimers and, consequently, leading to apoptosis. Induced apoptosis could be, thus, prevented by the addition of taxol, a drug that stabilizes the MTs (Bonfoco et al., 1995). Although many of the MT-stabilizing drugs, including taxol, do not cross the blood-brain barrier, recent advances of their nanosuspension delivery (Fan et al., 2021) or nasal administration of paclitaxel (Cross et al., 2021) appear to effectively overcome the brain blood barrier and accomplish neuronal cell-targeted drug delivery, thus, offering a potential for novel dementia therapeutic opportunities.

Protein aggregates in neurodegenerative diseases also contribute to neuronal loss (Chi et al., 2018). The localization as well as the composition of the aggregates differ from disease to disease [i.e., intraneuronal aggregates of tau protein in AD (Šimić et al., 2016) or aggregations of α-synuclein in Parkinson’s disease (Peng et al., 2018)]. In AD, tau protein undergoes posttranslational modifications (i.e., hyperphosphorylation) and/or truncation (Wischik et al., 1995), which is observed in NFTs. It is not clear, however, whether the phosphorylation occurs before the aggregation or after the formation of NFTs. Similarly, the debate whether tau aggregates or soluble tau oligomers are the toxic species is ongoing. Nonetheless, the NFTs may contribute to the activation of neuronal apoptotic mechanisms in AD (Nixon and Yang, 2011; Chi et al., 2018; Liu et al., 2020). Even though there are many papers in the literature that describe tau as a MT stabilizer which detaches from MTs when hyperphosphorylated (Chi et al., 2018), recent studies might call this statement into question (Baas and Qiang, 2019). Nonetheless, the fact that phosphorylation of tau hinders tubulin and MT assembly remains (Savastano et al., 2021). Furthermore, a study showed that tau protein is only bound to MTs for a very short time of 40 ms. The authors described that tau protein presented a “kiss and hop” interaction with tubulin molecules (Janning et al., 2014).

Not only have the NFTs been related to MT destabilization, but also Aβ. In fact, Fifre et al. (2006) described the possible role of Aβ in the degradation of different MAPs. Gevorkian et al. (2008) detected MT disruption in primary neurons exposed to Aβ as a result of the binding of Aβ with MAP1. In addition, modifications of MTs can also influence the ability of neurons to cope with Aβ neurotoxicity. Namely, MTs disruption leads to increased levels of cytotoxicity caused by the Aβ_1–42_ exposure, and *vice versa*, the stabilized MTs delay the toxicity caused by Aβ_1–42_ (Shamitko-Klingensmith et al., 2016). Similarly, in isolated neuronal cultures, Aβ oligomers cause tau-dependent MT breakdown mediated by spastin, an MT-severing enzyme (Zempel et al., 2013).

When analyzing VaD, it has been found that there is a loss neuronal cell volume in the dorsolateral prefrontal cortex (DLPFC) of patients with VaD when compared to controls (Mukaetova-Ladinska et al., 2009). Another study reported a selective loss of total tau protein in the temporal lobe of subjects with VaD that did not correlate with NFT, senile plaques or amyloid beta (Mukaetova-Ladinska et al., 2015). In a study by Gallart-Palau et al. (2015) where the levels of α1-tubulin and βII-tubulin isotypes were measured in VaD subjects and controls, no differences were observed in the temporal lobe. It is worth mentioning that the same study showed the tubulin proteins were significantly deamidated in VaD patients when compared to controls. Furthermore, these changes were not due to neurofibrillary pathology or any other lesion in VaD, i.e., visible infarcts (Gallart-Palau et al., 2015). Similarly, in a work done with human brain homogenates from VaD and AD patients, Mukaetova-Ladinska’s lab found a tendency for reduced levels of tubulin in the temporal lobe of VaD patients (Li et al., 2014), raising the question of whether changes in tubulin, i.e., posttranslational modifications, could be related to the observed loss of tau and neuronal volume.

Although there is no accumulation of NFTs in VaD, the temporal lobe appears to have a significant loss of soluble tau protein (Mukaetova-Ladinska et al., 2015), and this may result in the decreased neuronal cell volume followed by neuronal cell loss and apoptosis (Gemmell et al., 2012). The tubulin loss additionally may be result of: (1) neuronal death secondary to ischemic injury, (2) the process of diaschisis, (3) axonal injury in white matter and reduction of white matter volume, and (4) reduced dendritic arborization due to cell atrophy. Furthermore, these early changes in the interaction between microtubule associated proteins and tubulin may denote the neurobiological crossroad of further clinical and neurobiological progression to either AD or VaD. Having a better understanding of MTs and tubulin changes in the brain might lead to the development of an effective form of therapy.

## Microtubules

The cytoskeleton is a complex network of filaments that gives the cell its shape and mechanical resistance. In neurons, the cytoskeleton is formed by MTs, microfilaments and neurofilaments (Penazzi et al., 2016). Out of the three, MTs are the main protein filaments of the cytoskeleton and they are abundant in the cells (Horio and Murata, 2014; Burbaeva et al., 2020). They constitute approximately 10% of the total protein concentration in the brain (Cleveland et al., 1980).

MTs are involved in a variety of functions, such as cell motility, transport, cell shape and polarity, and mitosis (Mandelkow and Mandelkow, 1995). They are also necessary for synaptic plasticity, and their stability is essential for the physiological functioning of neurons (Carnwath et al., 2018). Cytoskeletal defects and altered MT-mediated processes are indeed linked to neurodevelopmental disorders, such as severe lissencephaly due to a mutation in the tubulin α1A gene (Kumar et al., 2010), or autosomal dominant disorders of axon guidance due to mutations in the TUBB3 gene (Tischfield et al., 2010).

A fundamental characteristic of MTs is that they are dynamic structures, which means they are able to alter their organization in order to adapt to changes in cellular shape (Goodson and Jonasson, 2018). This process, named “dynamic instability” (Horio and Murata, 2014), is considered an intrinsic property consisting on two opposed processes: polymerization or growth, and depolymerization or shrinkage, as seen in Figure 2B. The dynamic instability derives from the tubulin’s GTPase activity (Hyman et al., 1995; Nogales and Wang, 2006). Both polymerization and depolymerization can happen at the same time in the cell: whereas polymerization occurs in a temperature and concentration-dependent manner, depolymerization occurs randomly (Horio and Murata, 2014). Interestingly, this process occurs stochastically within the same filament, depending on whether MTs are bound to GTP or GDP, and both growing and shrinking MTs can be present in the same cell at any one given time. Thanks to this dynamic instability, fibroblasts are able to migrate and neurons extend their axon and dendrites (Mitchison and Kirschner, 1984; Brouhard, 2015).

MTs are hollow cylinders formed by heterodimers of α- and β-tubulin (Bryan and Wilson, 1971), with an approximate ratio of 1:1 (Alvarez et al., 1998). Both α- and β-tubulin monomers are very similar; they are ∼40% identical and 63% homologous (Nogales, 2013). Each tubulin monomer is composed of 450 amino acid residues which differ slightly from one another (Ludueńa et al., 1977; Sullivan and Cleveland, 1986), and a molecular mass of ∼50 kDa (Alvarez et al., 1998; Nogales et al., 1998). The monomers present some structural differences (Table 2). Due to the different functions MTs perform, they need to be specialized, and this specialization is achieved in two different ways (Gadadhar et al., 2017): **(a)** by interacting with microtubule binding proteins (MTBPs), or **(b)** by the tubulin code (Figure 2C), a highly important event in that it not only controls specific cellular functions, but it also has a role in human pathologies (Gadadhar et al., 2017). Moreover, the tubulin code is a mechanism by which MTs regulate themselves in yet another two different ways (Gadadhar et al., 2017):

**TABLE 2:** Structural differences between α- and β-tubulin (modified after Nogales et al., 1998; Nogales, 2013).

α-tubulin	β-tubulin
13% of alpha helices	13% of alpha helices
39% of beta sheets	42% of beta sheets
48% of random coils	45% of random coils
Asp -254 site at the E site, an ideal residue for the nucleotide hydrolysis	Lys-254 at the N site that strengthens the monomer-monomer interaction by interacting with the GTP phosphate group
GTP molecule always attached	GTP and GDP molecules are exchangeable for the polymerization of microtubules
Almost completely detyrosinated	Approximately 10% phosphorylated

1- **Expression of different isotypes of α- and β-tubulin**: both α- and β-tubulin have different isotypes that are encoded by different genes. In humans, there are 7α- and 8β-tubulin isotypes which differ in their C-terminal sequence and present tissue specificity (Ludueña, 1998; Fukushima et al., 2009; Ludueña, 2013). For example, α-tubulin Class II is mostly found in the testis, whereas α-tubulin III is found in brain and muscle (Ludueña, 2013). On the other hand, β-tubulin III is present only in neurons (Katsetos et al., 2003) whereas β tubulin Class VI is present mainly in hematopoietic cells (Murphy et al., 1987). Brains express α 1A-, α 1B-, α 1C-, α 4A-, and α8-tubulin, and β 2A-, β 3-, β 4-, and β5-tubulin (Fukushima et al., 2009). β-tubulin isotypes are evolutionary very well conserved and significantly better understood than α-tubulin isotypes (Ludueña, 2013). The level of expression of the isotypes in each specific tissue is very important since high βI-tubulin and low βII-tubulin levels have been described in breast cancer (Hasegawa et al., 2003; Ohishi et al., 2007). Furthermore, each isotype presents a specific function. Whereas βII is involved in neurite formation, βIII protects the MTs from reactive oxygen species (ROS) (Ludueña, 2013).
2- **Posttranslational modifications (PTMs) of tubulin (Table 3):** there are more than ten modifications that can occur at any given time (Fukushima et al., 2009; Janke, 2014; Gadadhar et al., 2017). More often than not, PTMs are found on stable long-lived MTs, such as neuronal, axonemal, and centriolar MTs (Janke, 2014). In modifying the tubulin, PTMs also modify the binding affinity of tubulin to MTBPs, such as tau, as it is observed by an increase in polyglutamylated tubulin (Eddé et al., 1990).

**TABLE 3:** Summary of main PTMs, the catalytic enzymes taking part in the reaction and the effects of PTMs on MTs/cells.

PTM	α-tubulin	β-tubulin	Catalytic enzymes	Effect on MTs
Acetylation	Yes	Yes	α-Tat1 and San acetyl transferase	Marker of stable MTs (Li and Yang, 2015) and recruitment of motor MTBPs (Dompierre et al., 2007)
Deacetylation	Yes	Yes	HDAC6 and Sirt2	Increases cell motility (Hubbert et al., 2002)
Tyrosination	Yes	No	TTL family	Marker of stable MTs
Detyrosination	Yes	No	VASH1/SVBP complex	Important for alignment of chromosomes during mitosis (Barisic et al., 2015)
Δ2-tubulin	Yes	No	CCP family	Marker of long-lived MTs (Paturle-Lafanechère et al., 1994)
Δ3-tubulin	Yes	No	CCP family	Marker of long-lived MTs (Paturle-Lafanechère et al., 1994)
Polyglutamylation	Yes	Yes	TTLL family	Regulation of MT-MAP interactions
Deglutamylation	Yes	Yes	CCP family	Regulation of MT-MAP interactions
Polyglycylation	Yes	Yes	TTLL family	Unknown in mammals
Deglycylation	Yes	Yes	Not found	Unknown in mammals
Polyamination	Yes	Yes	Transglutaminases	Stabilization of MTs in neurons
Phosphorylation			CDK1, Syk	Regulate MT behavior during cell division (Janke and Magiera, 2020)

### Interaction With Microtubule Binding Proteins

Any protein that binds to the MTs in a specified manner is considered a MTBP, and they are functionally categorized as stabilizers, destabilizers, capping proteins or bundler/cross-linkers (Goodson and Jonasson, 2018). Probably the most well-known MTBPs are the ones that comprise the MT associated protein (MAP) family, which includes tau protein and MAP2, the major MAPs in the brain (Melková et al., 2019), as well as MAP6, previously known as STOP.

1. **Tau:** this protein is enriched in axonal MTs, although it is also present in dendrites (Melková et al., 2019). Due to alternative splicing, there are six different tau isoforms, which differ in the number of MT-binding domain repeats (Fischer and Baas, 2020). Five of those isoforms weight around 40 kDa, and present multiple phosphorylation sites. When hyperphosphorylated, these form the characteristic NFTs observed in AD (Himmler et al., 1989), which hinder tubulin assembly (Savastano et al., 2021). On the other hand, the sixth isoform termed “Big Tau,” mainly expressed in the peripheral nervous system, weighs 100 kDa and does not present many phosphorylation sites; protecting it from aggregation into tangles (Fischer and Baas, 2020). In addition, the speculated function of big tau is the stabilization of mature axonal cytoskeleton (Oblinger et al., 1991).
2. **MAP2**: present in cell bodies and dendrites, MAP2 is considered a MT stabilizer (Goodson and Jonasson, 2018) as it increases MT rigidity (Felgner et al., 1997). Similar to tau protein, MAP2 is accumulated into granules, which leads to neurotoxicity and neuronal loss (Xie and Miyasaka, 2016). Loss of MAP2 has been widely linked to AD in human samples, as well as in cellular and animal models (Mukaetova-Ladinska et al., 2009; Baazaoui et al., 2017; Kandimalla et al., 2018; Manczak et al., 2018; Reddy et al., 2018; Beggiato et al., 2020; Liang et al., 2020).
3. **MAP6**: formerly known as STOP (stable tubule only polypeptide), it is a genuine MT stabilizer found predominantly in the axon, along with detyrosinated and acetylated tubulin; two PTMs present in stable MTs (Slaughter and Black, 2003).

### Posttranslational Modifications of Tubulin

Tubulin can undergo more than 10 different PTMs, some of them occurring solely on the tubulin, such as polyglutamylation. The major modifications that have been linked to neurodegeneration are acetylation, detyrosination, and polyglutamylation. In the following pages, we will discuss the tubulin PTMs and what it is known thus far about their role in neurodegenerative diseases.

1. ***Acetylation and Deacetylation:*** this modification is different from the others in that it happens in the lumen of MTs (Gadadhar et al., 2017). Acetylation can take place either on lysine 40 (K40) on α-tubulin (L’Hernault and Rosenbaum, 1985), where it is linked to stable MTs (Westermann and Weber, 2003; Wloga and Gaertig, 2010; Janke and Bulinski, 2011), or on K252 on β-tubulin (Janke, 2014), where it might slow down the incorporation of tubulin into MTs (Chu et al., 2011). The enzymes that catalyze the reactions are α-Tat1 (specific acetylation of tubulin) and San acetyl transferase (Akella et al., 2010; Janke, 2014), and HDAC6 and Sirt2 (involved in the deacetylation of tubulin and other substrates).
In a functional level, acetylated tubulin is implicated in intracellular trafficking, endoplasmic reticulum (ER) localization and ER-mitochondria interactions, as well as the regulation of MT dynamics (Daire et al., 2009). It has been observed that in most NFT bearing neurons there is a decrease in acetylated α-tubulin (Brion et al., 2001), a marker of stable MTs (Janke and Bulinski, 2011). Similarly, deacetylation is associated with dysfunctional MT-mediated axonal transport in neurodegenerative diseases, such as AD, Parkinson’s disease (PD) and Huntington’s disease (HD) (Li and Yang, 2015; Fernández-Barrera et al., 2018). In fact, HDAC6 (histone deacetylase 6, a multidomain cytosolic enzyme with α-tubulin deacetylase activity) is increased in AD (Zhang et al., 2013) and HD (Ferrante et al., 2003), and dysregulation of HDACs also take part in ischemic strokes (Fang et al., 2020).
2. ***Detyrosination and tyrosination:*** α-tubulin isotypes present a tyrosine residue at the end of the C-terminal sequence that β-tubulin isotypes lack; thus, rendering this modification specific to α-tubulin (Prota et al., 2013; Janke and Magiera, 2020). The tyrosine residue can be reversibly removed by the VASH1/SVBP complex (Aillaud et al., 2017) generating detyrosinated tubulin. Retyrosination of the tubulin, however, happens thanks to the tubulin tyrosine ligase (TTL) (Janke, 2014). These two modifications are spatially and temporally regulated. Whereas tyrosination is mostly found in the growing end of the axons in developing neurons (Ahmad et al., 1993; Sferra et al., 2020), detyrosination is mainly found in the dendrites of mature neurons, and it is considered a marker of stable MTs (Sferra et al., 2020).
Detyrosination regulates MT-MAP interactions whereas tyrosination has a role in spindle orientation and growth cone guidance in neuronal pathfinding (Magiera and Janke, 2014). In the context of AD, one study has suggested that the levels of tyrosinated levels are increased (Zhang et al., 2015).
3. *****Δ** 2-tubulin and **Δ** 3-tubulin:*** these terms are used when after detyrosination, additional C-terminal glutamate residues are eliminated from the tubulin sequence, which means that retyrosination is not possible (Janke, 2014; Gadadhar et al., 2017). Because of the lack of retyrosination, it is believed that one of the functions of Δ2-tubulin is to lock the tubulin in the non-tyrosinatable status or to reduce sites for polyglutamylation or polyglycylation (Magiera and Janke, 2014). It is estimated that 35% of α-tubulin in the brain corresponds to Δ2-tubulin (Paturle-Lafanechère et al., 1994). The enzymes that catalyze these reactions belong to the cytosolic carboxypeptidases (CCPs) family (Janke, 2014; Gadadhar et al., 2017). A study showed that there were increased levels of glutamylated Δ2-tubulin in the hippocampi of post-mortem patients of AD (Vu et al., 2017).
4. ***Polyglutamylation:*** first reported in 1990, it involves the addition of 1–12 glutamate units into a glutamic acid residue in the C-terminal region of either α- or β-tubulin (Eddé et al., 1990). In the brain tissue, there is an average of 3–6 glutamate residues on each tail, with as many as 11 and 7 residues detected on the α- and β-tubulin tails, respectively (Redeker, 2010). The polyglutamylation catalyzing enzymes belong to the TTL like (TTLL) family (Rogowski et al., 2010; Wloga and Gaertig, 2010) whereas the deglutamylases belong to the CCP family (Kimura et al., 2010). There are 8 members in the TTLL family that present different affinities for α- and β-tubulin, and different preferences for either initiating or elongating the glutamylation reaction, as shown in Table 4 (Yu et al., 2015). As for the CCP family, there are six members. CCP1, -2, -3, -4, and -6 shorten polyglutamylated chains, whereas CCP5 is the only monodeglutamylase found until date (Rogowski et al., 2010; Berezniuk et al., 2013).

**TABLE 4:** Family members of the TTLL family and their specific role in glutamylation.

	α-tubulin	β-tubulin
Initiates reaction	TTLL 1, 5, 6	TTLL 1, 4
Elongates chain	TTLL 6, 9, 11, 13	TTLL 7

Polyglutamylation is abundant in neurons, centrioles, basal bodies, axonemes of cilia and flagella, as well as in the mitotic spindle (Redeker, 2010), and it is implicated in the regulation of MT and MAP electrostatic interactions, since polyglutamylation affects the charges of the tubulin tails (Janke, 2014).
Maintaining the correct levels of polyglutamylation is essential. Several defects have been associated with either depletion or overexpression of the enzymes catalyzing glutamylation: TTLL1 knockout mice show respiratory problems (Ikegami et al., 2010), and TTLL6 knock out zebrafish show defective assembly of olfactory cilia (Pathak et al., 2007), whereas overexpression of TTLL6 causes ciliary defects in *Tetrahymena* (Suryavanshi et al., 2010). Another example comes from the Purkinje cell degeneration (*pcd*) mouse model, which lacks the CCP1 enzyme (Rogowski et al., 2010). These mice show overexpression of polyglutamylated tubulin and degeneration of Purkinje cells in the cerebellum (Magiera et al., 2018). Thus, balanced tubulin glutamylation levels are important for neuronal function and survival.
The excess of TTLL1-mediated polyglutamylation is the cause of cerebellar neurodegeneration in the *pcd* mouse model, and this neurodegeneration could be rescued upon depletion of TTLL1 (Magiera et al., 2018). The excessive polyglutamylation also impairs neuronal transport (Magiera and Janke, 2014), with the mutations of CCP1 in humans leading to childhood-onset neurodegeneration (Shashi et al., 2018). Of interest, in people with Alzheimer’s disease (AD), there is a reduction of α-tubulin and a tendency for increased polyglutamylation in the hippocampus when compared to age-matched controls (Zhang et al., 2015). It has already been shown that if tubulin is polyglutamylated with four or more glutamyl residues, the affinity to bind tau decreases (Eddé et al., 1990). However, little is known about MT-MAP interaction in neurodegenerative diseases and the implications this lower affinity has.
5. ***Polyglycylation:*** it refers to the generation of side chains of glycine residues within the C-terminal tails of α- and β-tubulin (Redeker et al., 1994). It happens at the same site as glutamylation and it is also catalyzed by TTLL enzymes (Rogowski et al., 2010). This PTM is restricted to cilia and flagella and the extent of the modification correlates with the length of the axonemes (Rogowski et al., 2010; Wloga and Gaertig, 2010). To date, there is very little known about the function of this modification in mammals. However, a link between the downregulation of TTLL3 and colon cancer has been described in humans (Rocha et al., 2014). Furthermore, it has been observed that polyglycylation in humans is not possible since the enzyme responsible for the elongation, TTLL10, is inactivated (Janke, 2014). There is little to no knowledge about the role of polyglycylation in neurodegeneration.
6. ***Polyamination***: for this modification, glutamine 15 (Q15) is considered to be the primary modification site, and the reaction is catalyzed by transglutaminases (Song et al., 2013). The reaction consists on the addition of amines to either α- or β-tubulin, adding positive charges to the acidic tubulin (Janke and Magiera, 2020). This PTM is irreversible and it most likely stabilizes microtubule subpopulations in neurons (Janke, 2014).
7. ***Phopshorylation:*** the Cdk1 enzyme catalyzes the phosphorylation on Serine 172 (S172) of β-tubulin (Fourest-Lieuvin et al., 2006), and it seems that this modification might be implicated in microtubule dynamics during cell division (Janke, 2014). Another tyrosine kinase known as Syk has been shown to phosphorylate an unidentified residue of α-tubulin (Peters et al., 1996).
8. ***Others:*** tubulin can also undergo palmitoylation (Caron, 1997), ubiquitination (Ren et al., 2003), glycosylation (Walgren et al., 2003), arginylation (Wong et al., 2007), methylation (Xiao et al., 2010), and sumoylation (Rosas-Acosta et al., 2005). However, there is not much information on these processes and how they affect disease pathogenesis.

## Therapeutic Drugs Targeting Microtubule Stability

### Microtubule Stabilizers

Several studies with MT stabilizers have been carried out as possible treatment for AD and other tauopathies:

1. **Epothilone D (BM2-241027):** this is a brain penetrant microtubule stabilizing agent able to polymerize tubulin and inhibit its depolymerization (Cheng and Huang, 2018). In a study following treatment with this drug, MT dynamicity decreased, whilst cognition improved, as shown in the Morris Water Maze task (Barten et al., 2012). Another study in cortical neurons put in display the importance of the dose, since it can affect the mitochondrial transport (Clark et al., 2020). Studies have also shown that BM2-241027 reduces axonal dysfunction, neurotoxicity, cognitive deficits, and AD-like pathology in PS19 aged tau transgenic mice (Zhang et al., 2012). Although in animal studies Epothilone D rescued working and spatial memory deficits in aged tau transgenic mice (reviewed in Yu et al., 2021), this drug was discontinued following a Phase 1 clinical study in 2013, with no information regarding drug’s effectiveness and side effects in humans (Bristol-Myers Squibb, 2013).
2. **TPI-287:** this CNS penetrating taxane was used in another clinical study to assess its efficiency as an AD treatment, progressive supranuclear palsy and corticobasal syndrome. Unfortunately, it was discontinued due to safety, i.e., severe anaphylactoid reactions (Tsai et al., 2020).
3. **Paclitaxel:** it is the generic name of taxol, a very common MT stabilizing drug approved by the Food and Drug Administration for the treatment of several types of cancer (Weaver, 2014). However, they evoke axonal degeneration (Gornstein and Schwarz, 2014). This neurotoxicity prevents it from being used in neurodegenerative diseases. However, its’ newer nasal formulation has been successfully used in transgenic animals. Cross et al. (2019) showed that intranasal paclitaxel administered once daily, prevented injury-induced memory deficits in mice. Furthermore, there was reduced evidence of axonal injury and synaptic loss (Cross et al., 2019). The same group has also shown that a transgenic mouse model of AD presented less tau-containing neurons in the CA1 and had improved memory after paclitaxel treatment (Cross et al., 2021). On the other hand, Lehrer and Rheinstein recently proposed that transdermal patches over the cervical spine could revolutionize drug therapy for AD, and may avoid the systemic side effects of the paclitaxel, such as anemia, leukopenia or peripheral neuropathy (Lehrer and Rheinstein, 2019).
4. **NAP (Davunetide):** this agent protects MTs against degradation induced by numerous MT disrupting agents, rendering it a possible potent drug against neurodegenerative diseases. The clinical trials performed in humans with tauopathies had no positive outcome (Ivashko-Pachima and Gozes, 2021). In a phase II double-blind randomized controlled trial, NAP showed cognitive and functional improvement in mild cognitive impairment (MCI) patients, after a 12-week intranasal NAP administration (reviewed by Yu et al. (2021). By the time this review is being written, clinical trials on MCI, progressive supranuclear palsy and schizophrenia have all been discontinued, and the one for frontotemporal dementia is inactive (as per alzforum webpage 20.09.2021).

As observed in these three examples, MT stabilizers have severe side effects (Chiorazzi et al., 2009; Brandt and Bakota, 2017), and although they might help in neurodegenerative diseases, they require very fine-tuning to avoid toxicity.

### Drugs Targeting Tubulin Posttranslational Modifications

The only tubulin PTM that has been targeted thus far has been acetylation. There are several histone deacetylases (HDACs) which deacetylase α-tubulin, such as HDAC6 and SIRT2 (Hubbert et al., 2002; North et al., 2003). There are studied that show prevention of cognitive decline upon inhibition of SIRT2 (Diaz-Perdigon et al., 2020). Positive effects were also observed with HDAC6 inhibitors (Selenica et al., 2014). Vorinostat is an HDAC inhibitor which tolerable doses is being studied in patients with mild AD (clinical trial identifier: NCT03056495).

For a more detailed review on cytoskeleton-targeted drugs, refer to Eira et al. (2016).

### Drugs Targeting Necroptosis May Stabilize Microtubule

Necrostatin-1 (Nec-1) is an anti-necroptotic molecule that directly targets Aβ and tau proteins, alleviates brain cell death and ameliorates cognitive impairment in AD models (Yang et al., 2017). Via targeting and reducing both Aβ oligomers and hyperphosphorylation of tau protein, it may also improve the MT stabilization and may serve an important role in the development of preventive approach for AD. These findings come from an animal APP/PS1 model (Yang et al., 2017), and have been recently extended to a vascular animal model with bilateral common carotid artery stenosis. Namely, in the latter vascular animal model, Nec-1 improved animal behavior and enhanced the inhibitory effect of environment enrichment on inflammation response (Zhang et al., 2019).

Another necroptosis inhibitor, necrosulfonamide (NSA), has also been investigated in a rat model of AD. Administration of NSA intraperitoneally for 6 weeks alleviated phosphorylated tau protein and Aβ accumulation, and regulated the high hippocampal expression of tumor necrosis factor-alpha (TNF-α), β-site amyloid precursor protein cleaving enzyme 1 (BACE1), glycogen synthase kinase-3β (GSK-3β), and acetylcholinesterase (Motawi et al., 2020). NSA has been identified as a novel promising anti-AD treatment via targeting necroptosis, and possibly indirectly contributing to MT stabilization in AD.

All the evidence for the necroptotic agents in AD treatment comes from animal studies, and no human clinical trials are being conducted Similarly, the evidence of these agents upon the MT network, as a result of their effect upon hyperphosphorylated tau protein and Aβ, is still missing.

## Conclusion

AD and VaD represent almost 85% of all clinically diagnosed dementia cases, percentage that raises if we take mixed dementia into account, and neuronal loss is a hallmark in all of them. The neuronal loss is not only the best predictor for cognitive decline, but in AD and VaD it also correlates with the severity of the disease, as well as with Braak stages (Buchanan et al., 2020). On a morphological level, neuronal loss results in significant changes in the cytoskeleton of the cells, and consequently, in the MTs. Stability of MTs is, thus, essential for the physiological functioning of neurons.

Tau and MAP2 are MTBP involved in regulating the dynamics and assembly of MTs. In AD, tau is hyperphosphorylated and leads to neuronal loss. Polyglutamylation also regulates the interaction between tau and tubulin, where the more polyglutamylated the tubulin is, the less affinity it has to binding tau, ultimately leading to destabilization of MTs (Eddé et al., 1990). Although this was shown already in 1990, we still don’t know what exactly happens to MT-MTBP interactions in neurodegenerative diseases. Understanding the interplay between tubulin, its PTMs and the binding affinity to MTBPs may help determine if there is a specific event that can prevent or stop neurodegeneration.

Maintaining the levels of tubulin polyglutamylation has proven to be essential for neuronal survival. In fact, it has been shown that there is a tendency for increased α-tubulin polyglutamylation in AD brains when compared to controls (Zhang et al., 2015). However, the number of subjects in this study was relatively low, and a larger number of brain samples should be studied. In addition, tyrosinated levels of tubulin were also measured, with the same outcome. Increasing the number of subjects in these studies is very important. Determining whether polyglutamylation and tyrosination play a role in AD and VaD could have clinical relevance that might lead to the development of new and efficient dementia treatments. It may be possible that tubulin plays a more important role than we know in these diseases, and that its stabilization and PTMs could have a big impact on the severity and progression of the diseases.

Even though there is no increase of phosphorylated tau protein in VaD patients, there is a decrease in the levels of total tau protein (Mukaetova-Ladinska et al., 2015). It is possible that changes in tubulin, such as an increase in polyglutamylation, may lead to a decreased affinity for tau, changes in MT dynamics and increasing the ratio of shrinkage to growth. This could be one plausible explanation for the observed neuronal loss in VaD (Gemmell et al., 2012). It is worth mentioning that some authors state tau works through a “gain of toxicity” mechanism, and that tau-lowering levels might be beneficial in AD and other tauopathies (Goedert, 2016). However, we do not speculate that a decrease in tau could lead to MT destabilization, but that the opposite might happen: tubulin PTMs could lead to molecular changes in tau that, ultimately, could take part in the destabilization of MTs. Even though there is no much literature linking VaD with axonal transport disruption, there are a couple of studies that do. One of them suggests that compromised axonal transport might play an important role in a type of VaD (Binswanger’s disease) (Akiguchi et al., 1997). The other one states that since white matter is often affected in VaD, likely both demyelination and focal axonal injury occurs in VaD (Ihara et al., 2010). Therefore, it is fundamental for in-depth studying of VaD to understand the molecular mechanisms as well as the MT-MTBP interactions to aid the pharmacological treatment of VaD.

It is unclear whether MT abnormalities have a causal and early role in the disease process or represent a common end point downstream of the neurodegenerative cascade. Their presence in absence of overt Aβ and tau pathology, as it is the case in VaD, would argue that they may be an early change, though the MT destabilization as seen in AD argues for the opposite. Understanding the chronology of this triad of tightly woven molecular events will provide a new window of opportunity to arrest the dementia process in early, preclinical stages. It is intriguing to speculate that the chronology of events may differ among different dementia syndromes, and thus drive the design of novel dementia-specific therapies.

Restoring the MT network and the balance between labile and stable MTs might be fundamental to stop the progression of cell death and neurodegeneration. We believe that regulation of MTs by the different PTMs might be key on determining if and with how much affinity does tubulin bind to MTBPs. These changes might not affect the cells immediately, but they might accumulate over time and have a progressively negative impact on the brain. What if tubulin is the key link that is missing? What if the accumulation of proteins and gradual synaptic loss comes from a previous gradual accumulation of tubulin PTMs? What if these accumulations or sudden increase in some of the PTMs lead to a strong enough cell destabilization that produces the neuronal loss in neurodegenerative diseases? These are only some of the questions that may help guide the development of the new generation anti-dementia treatments.

## Author Contributions

ES-M wrote the article. RL-C, FG, RK, and EM-L contributed to the discussion, reviewed, and edited the manuscript. All authors contributed to the article and approved the submitted version.

## Conflict of Interest

The authors declare that the research was conducted in the absence of any commercial or financial relationships that could be construed as a potential conflict of interest.

## Publisher’s Note

All claims expressed in this article are solely those of the authors and do not necessarily represent those of their affiliated organizations, or those of the publisher, the editors and the reviewers. Any product that may be evaluated in this article, or claim that may be made by its manufacturer, is not guaranteed or endorsed by the publisher.
